# Mini-Review on the Enzymatic Lipophilization of Phenolics Present in Plant Extracts with the Special Emphasis on Anthocyanins

**DOI:** 10.3390/antiox11081528

**Published:** 2022-08-05

**Authors:** Karina Jasińska, Agata Fabiszewska, Ewa Białecka-Florjańczyk, Bartłomiej Zieniuk

**Affiliations:** 1 Department of Food Engineering and Process Management, Institute of Food Sciences, Warsaw University of Life Sciences (WULS-SGGW), 159c Nowoursynowska St., 02-776 Warsaw, Poland; 2 Department of Chemistry, Institute of Food Sciences, Warsaw University of Life Sciences (WULS-SGGW), 159c Nowoursynowska St., 02-776 Warsaw, Poland

**Keywords:** plant extracts, lipophilization, phenolic compounds, lipase, anthocyanins, esterification

## Abstract

Different plant extracts have the potential to be important sources of phenolic compounds. Their antibacterial, antifungal and antioxidant properties are of interest to researchers due to various possibilities for use in the pharmacy, cosmetic and food industries. Unfortunately, the direct application of phenolics in food is limited because of their hydrophilic nature and low solubility. The review is devoted to the recent advances in the methods of lipophilization of phenolic extracts along with the use of enzymes. The concept of extract modification instead of single compound modification is based on the expected synergistic effect of many phenolic compounds. The main focus is on the phenolic compounds found in fruits, flowers and leaves of different common and underutilized as well as medicinal, folk-medicinal or endemic plants. The compiled papers point to the great interest in the modification of anthocyanins, highly active but often unstable phenolics. Some examples of other flavonoids are also outlined. The possible applications of the lipophilized plant extracts are presented for improving the stability of edible oils, decreasing the content of acrylamide, exhibiting higher color stability in thermal processing and increasing the nutritional value.

## 1. Introduction

**Figure 1:**
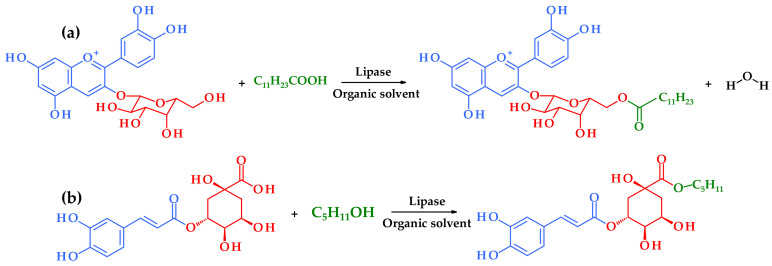
Enzymatic synthesis of (**a**) cyanidin-3-*O*-(6″-dodecanoyl)galactoside, and (**b**) pentyl chlorogenate. The phenolic moiety is highlighted in blue, sugar moiety and quinic acid are in red, and lipophilic molecules (fatty acid/alcohol) are marked in green.

Plant extracts have been used since ancient times. People were using phytonutrients and biologically active compounds long before they were described and their structures were discovered. Nowadays, it is assumed that approximately 20% of known plants have been applied in pharmaceutical studies and have positively affected human health [1]. Many plant origin substances have well-described antifungal, inflammatory, antibacterial and antitumor properties. It is well known that many plants are abundant in polyphenolic compounds; therefore, plant extracts are also a good source of these secondary plant metabolites. For example, vegetables, fruits, nuts, seeds, stems, flowers and roots are plentiful in polyphenols. To date, there are more than 8000 polyphenolic structures known, and for many years, research on their functional and therapeutic properties has been carried out [2]. The diversity in the quantity and specific structures of phenolics is observed between plants. They are a large and diverse group, including flavonoids, e.g., flavonols, flavanols, anthocyanins and nonflavonoids, e.g., phenolic acids (hydroxycinnamic and hydroxybenzoic acids), lignans and stilbenes [3]. Phenolic compounds are of general interest because of their ability to be very effective in free radical scavenging in foods. They can easily donate hydrogen or electrons to convert free radicals into more stable and safe compounds [4]. Food technologists are trying to fortify groceries with phenolics which can serve as antioxidants, but their use in lipophilic matrices is limited due to their hydrophilic nature and low solubility. It is necessary to derive them into the more amphiphilic form which let them keep their original functional properties. The improvement can be made by a modification defined as lipophilization. This term encompasses chemically or enzymatically esterifying the appropriate group of a phenolic compound with a fatty alcohol or a fatty acid [4,5]. In the case of phenolic acids, this will be esterification with aliphatic alcohols. Flavonoids, which are usually present in nature in the form of glycosides, can be lipophilized by esterification of the primary (predominantly) alcohol group in the sugar molecule with fatty acids. It should be noted that this reaction does not concern phenolic groups and therefore should not adversely affect the antioxidant potential of the esterified compounds (Figure 1). The esterification of phenolic compounds can be performed by chemical or enzymatic methods. The latter is especially recommended because the use of biocatalysts allows for applying milder conditions, assures selective specificity and minimizes side reactions and formation of by-products. Enzymatic reactions are more environmentally friendly because of the reduced amount of energy consumption and waste material production. Sometimes these two methods are applied alongside in chemoenzymatic lipophilization, which can be carried out in two ways: under solvent or solvent-free conditions [6].

Improving the solubility of phenolics in organic media is performed often as a single compound modification [5,7]. However, plant extracts have been found to generally show better antioxidant properties than most pure phenols, which may suggest a synergistic interaction of antioxidants with each other [8].

Using whole plant extracts, which contain different kinds of phenolic compounds, and verifying their synergistic effect is still a novel approach. It is obvious that extracts from the individual types of fruits differ in the content of various antioxidants and their antioxidant activity is related to the content of the type of individual phenolic compounds. Therefore, the method of lipophilization depends on the composition of the extract [9].

This review aims to summarize the current knowledge concerning enzymatic lipophilization of plant extracts. Recent advances in the methods of enzymatic modification of phenolic extracts and investigated enzymes as well as exerted biological activities, including nutritional and pharmaceutical applications, are discussed. In the paper, we present results for enzymatic modifications of phenolic compounds occurring in multi-compound mixtures (whole-plant extracts or extracts from parts of plants) as well as purified mono-component phenolic extracts. The main group of phenolic derivatives via enzymatic lipophilization are anthocyanins and other flavonoids.

## 2. Lipophilization of Extracts from Fruits

Extracts of fruits such as grapes, raspberries, cherries, blackberries, blueberries, apples or rowan are characterized by a wide spectrum of antioxidant properties and contain many beneficial substances, especially polyphenols, which are one of the essential groups of compounds in secondary metabolism.

Rowan (*Sorbus aucuparia* L.) is certainly an interesting source of phenolic compounds. The ripe fruits of rowan are spherical with orange to red color and tart-bitter in taste. The major constituent of rowan phenolic extract is chlorogenic acid, which is amounted to approximately 80% of phenolics. In smaller quantities, there are neochlorogenic and feruloylquinic acids present, which are isomers and derivatives of chlorogenic acid, respectively [10].

**Figure 2:**
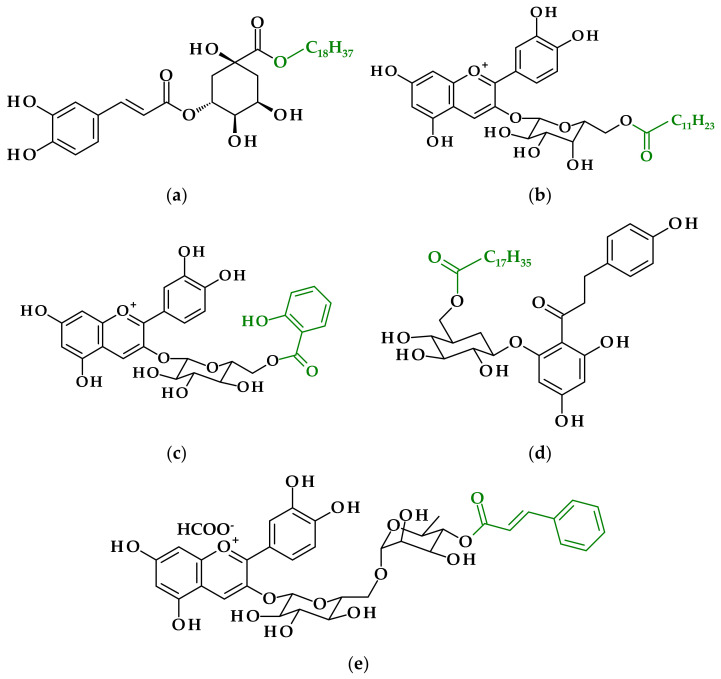
Phenolic derivatives from lipophilized extracts of fruits: (**a**) octadecyl chlorogenate derivative of chlorogenic acid-depside of caffeic acid and quinic acid [10]; (**b**) cyanidin-3-*O*-(6″-dodecanoyl)galactoside derivative of anthocyanins (glycosides of anthocyanidins) [11]; (**c**) cyanidin-3-(6-salicyloyl)glucoside–derivative of anthocyanins (glycosides of anthocyanidins) [12]; (**d**) phloridzyl octadecenoate-derivative of dihydrochalcone glucoside [13]; (**e**) cyanidin-3-*O*-(4‴-cinnamoyl)rutinoside derivative of anthocyanins (glycosides of anthocyanidins) [14].

Due to the poor lipophilic character of rowan phenolic extract, Aladedunye et al. [10] changed its lipid solubility by lipase-catalyzed esterification of the carboxyl group of the phenolic acid with aliphatic alcohol-octadecanol. In the enzymatic reaction octadecyl chlorogenate (Figure 2a) was obtained and subsequently was evaluated as an antioxidant agent in frying and storage tests.

The presence of chlorogenic acid in rowan phenolic extract, and more specifically a catechol ring, resulted in an antioxidant activity and increasing the oxidative stability of oils. In the storage test, both purified natural extract (PNE) and its lipophilized form (LE) inhibited the primary oxidation, which was observed by lower peroxide values compared with the control (oil without addition of any compounds). Interestingly, there were no significant differences between PNE and LE. On the contrary, the incorporation of the octadecyl chain to the structure of chlorogenic acid impacted on oil protection during the deep frying of potato chips. Additionally, in comparison with the control and non-modified rowan extract formation of polar components and di- or polymeric triacylglycerols was remarkably inhibited. The abovementioned paper suggests the feasibility of developing functional antioxidants with the use of wild/ornamental edible fruits [10].

Fruits can stand as a good source of anthocyanins, which are glycosides of anthocyanidins that are based on the flavylium cation with hydroxyl and methoxyl groups substituted for its hydrogen atoms. The vast majority of anthocyanins are derived from six aglycones: cyanidin, delphinidin, malvidin, pelargonidin, peonidin and petunidin [11]. Anthocyanins are the main water-soluble class of flavonoids and occur in vegetables such as red onion, red cabbage and sweet potatoes, as well as in fruits, e.g., strawberries, raspberries and cranberries [15]. They are also present in flowers and can absorb visible light. Those natural pigments are responsible for flower or other plants’ color with the range from red to blue, which result in using them as described by the authors of [16]. The unique properties of described compounds are attributed to their structures. They have shown diverse biological activity such as antioxidant, antimicrobial and anti-inflammatory properties; moreover, they have played a role in cardiovascular protection and obesity prevention, and are capable of decreasing serum triglyceride [11,14,15]. Nonetheless, the use of anthocyanins is limited because their instability and hydrophilic properties restrict their application in lipidic media. Mentioned compounds are sensitive to pH values, light, heat or ion concentration. The physiological activity is also hindered due to the poor solubility in fat, that they cannot pass through the lipid bilayer [17]. Anthocyanin stability can be improved, for example, through esterification that can be achieved by chemical or enzymatic methods. Definitely, the latter is performed in mild conditions and with high selectivity, without hazardous catalysts or appearing as by-products [14,16].

In the research by Yang et al. [11], anthocyanins isolated from alpine bearberry (*Arctostaphylos alpina*) were enzymatically acylated through hydroxyl group in sugar moiety with lauric, myristic, palmitic and stearic acids, respectively, with the use of *C. antarctica* lipase B (CALB) as a catalyst. A saturated fatty acid with 12 carbons proved to be the best acyl donor to synthesize ester of cyanidin-3-*O*-galactoside (Figure 2b) and the conversion rate amounted to 73% when *tert*-butanol was a solvent, the temperature was 60 °C for 72 h and the molar ratio of anthocyanin to lauric acid was 1:10.

Interestingly, the introduction of lauric acid into the cyanidin-3-*O*-galactoside structure improved thermo-stability as well as lipophilicity by determining the octanol/water partition coefficient (logP). Comparison of antioxidant activity of cyanidin-3-*O*-galactoside and its lauric ester assessed by DPPH· and FRAP assays revealed that acylation only slightly affected the antioxidant capacity, and activity of lauric ester measured in the hydrophilic system was largely retained [11].

Blackcurrant (*Ribes nigrum* L.) is plentiful in anthocyanins, which are natural colorants and bioactive ingredients. Using them as fruit extracts has some barriers to being successfully applied in food, cosmetic and pharmaceutical industries because of the hydrophilic nature of phenolic compounds. Cruz’s group [18] tried to improve the lipophilicity and enlarge the application of blackcurrant anthocyanins by enzymatic acylation using *Candida antarctica* lipase B. They extracted anthocyanins from waste blackcurrant (*Ribes nigrum* L.) fruit skins which contained rutinosides and glucosides of cyanidin and delphinidin and used them as a substrate in reaction with octanoic acid. The research showed that cyanidin and delphinidin glucosides were acylated, whereas the rutinosides were not. This was probably caused by the absence of a primary hydroxyl group in the disaccharide moiety. The lipophilization method was proposed as a new technique to separate anthocyanins with different glycosylation patterns in mixtures otherwise difficult to separate [18]. Furthermore, Yang et al. [19] enzymatically acylated anthocyanin-rich fractions isolated from blackcurrant (*Ribes nigrum* L.) with lauric acid. They successfully monoacylated delphinidin-3-*O*-glucoside, delphinidin-3-*O*-rutinoside, cyanidin-3-*O*-glucoside and cyanidin-3-*O*-rutinoside showing that there is a possibility to acylate rutinose moieties of anthocyanins. In the case of rutinosides, esterification occurred at 4-OH of the rhamnose unit. According to the authors, the discrepancy with the Cruz results comes from using different reaction conditions and acylating agents. Conducted reactions enhanced lipophilicity and thermostability, as well as improved the inhibition of lipid peroxidation in a lipophilic environment. Yang et al. [19] noticed also that more hydrophilic anthocyanin rutinosides after esterification were more lipophilic than glucosides of the same anthocyanidins [19].

Raspberry (*Rubus* L.) belongs to the same group of fruit as blackcurrant so it is very rich in anthocyanins. Therefore, Teng et al. [12] acylated anthocyanins present in an extract from raspberry with methyl salicylate via lipase-catalyzed reaction under reduced pressure and achieved a conversion rate of 84.26%. The product of the reaction was cyanidin-3-(6-salicyloyl)glucoside, and the few analyses of it were obtained (Figure 2c). The researchers found that enzymatic acylation could be a helpful way for developing the processing stability of anthocyanins and also to maintain their powerful antioxidant capacity [12].

Stevenson et al. [20] chose a blueberry extract and apple extract. They used immobilized *Candida antarctica* lipase B to acylate flavonoid glycosides from fruit with carboxylic acids. Depending on the kind of acyl donors (palmitic, cinnamic and phenylpropionic acids) the conversions ranged from ~25% to ~95%. The enzymatic lipophilization of tested extracts, which were a good source of flavonoids, presented strong selectivity for acylation of glucosides with a primary aliphatic hydroxyl group on the sugar moiety, such as phloridzin and anthocyanidin glucosides and galactosides. For compounds lacking a primary aliphatic hydroxyl group, such as flavonoid aglycones, chlorogenic acid or anthocyanidin arabinosides, no acylation was noticed. They observed that acylation, especially with palmitic acid, enhanced lipophilicity and solubility in the reaction medium [20].

Aladedunye and Matthäus [13] also tried to take advantage of apple extract. They used it for the study of *Canadian crabapple* and reported that the major polyphenolic component of the extract was phloridzin, which includes seven hydroxyl functional groups, but only the primary aliphatic group in glucose moiety was active. Enzymatic acylation was carried out with octadecanoic acid by *Candida antarctica* lipase B. The phloridzyl octadecanoate (Figure 2d) was a product of the reaction and after lipophilization was added to rapeseed oil to assess the antioxidant activity of the modified extract during storage and frying. Results showed that attachment of the octadecyl tail enhanced the antioxidant performance of phloridzin in rapeseed oil during the frying of potato chips but had no relevant impact on the oxidative stability of the oil under storage conditions [13].

The significant potential of grape seed was discovered by Chen and Yu [21]. Proanthocyanidins, which are major polyphenol components in grape seed, were modified structurally to improve their lipophilicity using esterification catalyzed by immobilized lipase Lipozyme TL IM with lauric acid. The outcome showed that the conversion rate was 84.1%. The presence of GSP (grape seed proanthocyanidin) derivatives was confirmed by HPLC-MS-MS. It was found that GSP derivatives had higher lipophilicity than GSP and gained the highest DPPH radical scavenging activity compared to GSP, BHT (butylated hydroxytoluene) and TBHQ (*tert*-butylhydroquinone). The researchers also proved that GSP derivatives were nontoxic so this indicated its potential addition to food as an oil-soluble antioxidant. It is worth underlining that the authors used Lipozyme TL IM as a biocatalyst instead of the commonly used CALB. Moreover, it was unusual that there were esterified hydroxyl groups present in the phenolic ring and despite the lower number of hydroxyl groups in the modified compound, the antioxidant effectiveness was higher than GSP [21].

The work of Fernandez-Aulis et al. [14] showed a study on the preparation of a series of acylated anthocyanins from five purified extracts from underutilized plants. Among the series of reactions carried out, several of them clearly differ from the above-cited papers. Cyanidin glycoside from trueno fruits (*Ligustrum japonicum*) was subjected to transesterification with vinyl cinnamate and cyanidin-3-*O*-(4‴-cinnamoyl)rutinoside (Figure 2e) and was synthesized at a 45.5% conversion yield [14]. The usage of vinyl esters in the enzymatic reactions is a well-known way to increase the conversion yield due to the shifting of the equilibrium state towards the formation of the product because of the tautomerization of vinyl alcohol to acetaldehyde [22].

The fruits mentioned above are well known because of their attractive biofunctional properties and high content of flavonoids and other polyphenols. However, other examples that are not as popular as the berry fruit group, but share the same features, can be found on the planet. In the first instance, jaboticaba (*Myrciaria cauliflora*) can be provided, which easily grows in tropical regions of the planet, especially in Brazil, but also in Argentina, Bolivia, Paraguay and Peru. De Castro et al. [23] extracted polyphenols from the skin of jaboticaba fruits and then performed enzymatic acylation using palmitic acid as an acyl donor and lipase B from *Candida antarctica* (CALB) as a biocatalyst. The researchers analyzed antioxidant properties and content of the fraction of phenolic compounds. They detected the presence of ellagic acid, quercetin, rutin, delphinidin-3-glucoside and cyanidin-3-glucoside and confirmed that the activity of the jaboticaba peel extracts were at least as high as those reported in the literature for several more known fruits. Due to enzymatic modification, it was possible to obtain two anthocyanin derivatives, namely, delphinidin-3-*O*-(6″-palmitoyl)glucoside and cyanidin-3-*O*-(6″-palmitoyl)glucoside [23]. The second example can be jambolan (*Syzygium cumini*), a tropical, edible fruit with a deep purple peel when ripe, native to the Indian subcontinent. It has an attractive color due to the presence of anthocyanins (mainly delphinidin-3,5-diglucoside and petunidin-3,5-diglucoside [24], especially in its peel part and is a novel source of natural colorants for the food system. For this reason, Sari et al. [25] tried to acylate anthocyanins of jambolan fruit. They enzymatically synthesized derivatives of phenolic compounds by lipase-catalyzed transesterification reaction with cinnamic acid. The results showed that acylation with chosen acid gave a change of color from red to purplish-red in the beverage model system, pH = 3 and increased thermal and light stability of modified anthocyanins. Unfortunately, enzymatic lipophilization partially declined antioxidant activity [25]. The data on the use of fruit extracts in the synthesis of lipophilic antioxidants are summarized in Table 1.

## 3. Enzyme-Assisted Derivatization of Phenolics Present in Flower Extracts

Flower extracts are also a natural source of bioactive compounds providing, for example, antioxidant or anti-inflammatory properties, but focus on them has been as natural dyes, widely used to produce natural cosmetics and foods. Plant pigments are classified into four main categories: chlorophylls, anthocyanins, carotenoids and betalains. Among them, anthocyanins are of particular interest and colored anthocyanin pigments have been traditionally used as natural food colorants. Unfortunately, as mentioned in the case of fruit anthocyanins, the color and stability of these pigments are influenced by pH, light, temperature and structure. In acidic conditions, anthocyanins appear red but turn blue when the pH increases. Therefore, both solubility and resistance to changes under acid pH are important [26,27].

The possibility of increasing the lipophilicity of anthocyanins derived from flower extracts was studied by Marquez-Rodriguez et al. [16]. Delphinidin 3-*O*-sambubioside was extracted and purified from *Hibiscus sabdariffa* flowers, and then was subjected to enzymatic acylation with octanoic acid. Due to the hydrolysis occurring in the disaccharide residue, the reaction conditions had to be optimized. Two solvents, the ratio of substrates, enzyme concentrations and three different counter-ions, were evaluated. The highest conversion yield (15%) with no observed hydrolysis was achieved when 2-methyl-2-butanol was a solvent, and delphinidin 3-*O*-sambubioside was applied with formate as counter-ions.

The physicochemical characterization of the new synthesized lipophilic pigment, such as partition coefficient and color properties, were investigated. Acylation of delphinidin 3-*O*-sambubioside improved the lipophilicity of the obtained compound, as well as improved the stability by hydration resistance and presented a stabilization of the quinoidal base, one of the equilibrium forms of flavylium cation, responsible for blue color at a neutral or moderate alkaline pH. The presented results show the possibility of synthesizing and the potential use of natural-based lipophilic pigments in different industries [16].

Marathe et al. [15] observed that flower petal waste is generated in tremendous quantities in places of worship, and such waste is usually disposed of in wastelands. Rose petals occurred more often. Roses are definitely a good source of anthocyanins, and due to their low stability and sensitivity to different conditions, the authors proposed carrying out esterification reactions as an idea to partially reduce the formation of organic waste and improve the physicochemical properties and biological activity of flavonoid compounds present in rose petals.

Similar to other authors, lauric acid was used as an acyl donor, and molar conversion of 63% of anthocyanins to laurate esters was reached when the temperature of 40 °C, acetonitrile, a molar ratio of 1:100 (cyanidin to a fatty acid), *C. antarctica* lipase, and molecular sieves were used. Isolated anthocyanins and their lauric acid esters were compared in the DPPH^•^, ABTS^•+^ and FRAP assays to assess their antioxidant activity. Three mentioned tests revealed that antioxidant activity of acylated anthocyanins was significantly reduced in comparison with anthocyanins that were not subjected to esterification, but it can be linked to the higher lipophilic character of the obtained esters as well as partial degradation of anthocyanins during the reaction. The use of anthocyanin lauric acid esters was studied as a colorant in cupcakes and in filling cream to sandwich biscuits as well as a pre-extrusion colorant in puffed rice extrudates. Likewise, to their precursors, the color of laurate esters of anthocyanins was pH-dependent, which was confirmed at varying pH using a colorimeter and in the color of cupcakes. Better thermo-stability of biscuit cream filling was also observed when the products of esterification were used compared to native anthocyanins and synthetic colorants. Similar observations were provided by the authors in extrusion experiments, and the laurate esters exhibited higher color stability in thermal processing. Enzymatic esterification of anthocyanins from rose petals and confirmation of their better thermal stability and other properties may in the near future affect their consideration as natural-based colorants in the food industry [15].

**Figure 3:**
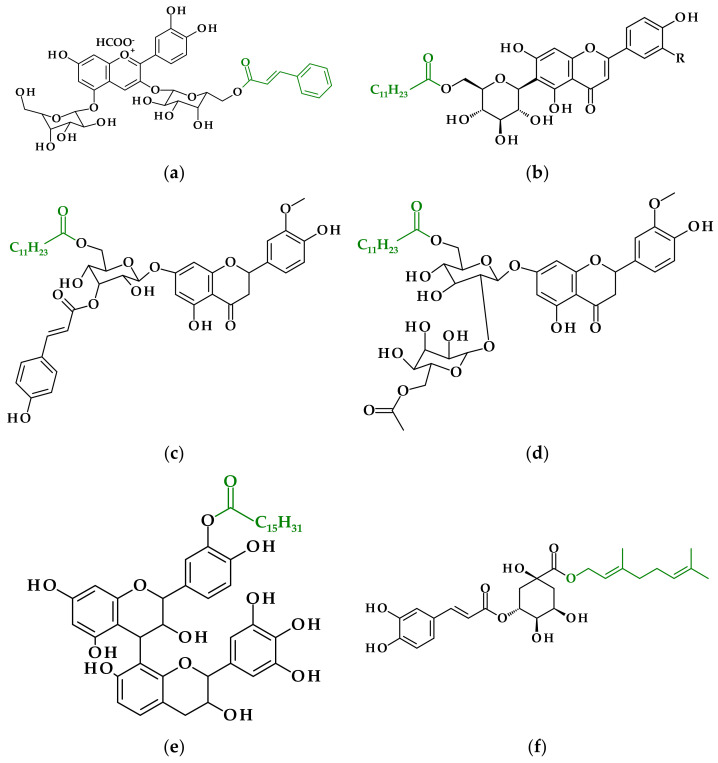
Phenolic derivatives from lipophilized plant extracts other than fruits extracts: (**a**) cyanidin-3-*O*-(6″-cinnamoyl)glucoside-5-*O*-glucoside-derivative of anthocyanins (glycosides of anthocyanidins) [14]; (**b**) isoorientin-6″-laurate/isovitexin-6″-laurate-derivative of flavonoid C-glycosides [28], isoorientin: R = OH, isovitexin: R = H; (**c**) laurate ester of chrysoeriol-7-*O*-β-D-(3″-*E*-*p*-coumaroyl)-glucopyranoside-derivative of monosaccharidic flavone [29]; (**d**) laurate ester of chrysoeriol-7-[6‴-*O*-acetyl-β-D-allosyl-(1→2)-β-D-glucopyranoside]–derivative of disaccharidic flavone [29]; (**e**) procyanidin and prorobinetinidin heteroduplex esterified with palmitic acid–derivative of proanthocyanidin [17]; (**f**) geranyl chlorogenate–derivative of chlorogenic acid [30].

Fernandez-Aulis et al. [14] acquired cyanidin glycosides from bottlebrush (*Callistemon citrinus*) flowers from trees growing in the streets of Mexico City, which are also used in traditional medicine. It may be that the content of isolated anthocyanins was not very high, namely 6.15 ± 0.39 g/g DW for bottlebrush flower, but cyanidin-3-*O*-(6″-cinnamoyl)glucoside-5-*O*-glucoside was obtained (Figure 3a). In the enzymatic reactions, vinyl cinnamate and cinnamic, dihydrocinnamic, dihydroferulic and dihydrosinapic acids were used as acyl donors, and some new acylated derivatives were obtained with improved antioxidant activity and thermostability, in comparison to the cyanidin-3-glucoside [14]. Enzyme-assisted derivatizations of phenolics present in flower extracts are summarized in Table 2.

## 4. Lipophilization of Extracts from Plant Leaves

Enzymatic acylation was applied to improve solubility in lipidic matrices or lipophilic media of flavonoids present in the bamboo-leaf extract. Ma et al. [28] focused on the enzymatic acylation of two flavonoids from bamboo-leaf extract-isoorientin and isovitexin, with three fatty acids as acyl donors (lauric C12, myristic C14 and palmitic C16 acids). The acylation in the presence of CALB occurred at the primary hydroxyl group of glucose moiety and only monoesters were detected. The highest conversion yields exceeding 75% were achieved when lauric acid was used in *tert*-amyl-alcohol as the reaction medium. The partition coefficients of acylated derivatives, which are attributable to their lipophilicity, increased with elongation of the acyl chain length [28]. Palmitoyl isoorientin ester was also synthesized via lipase-catalyzed esterification, whereby the product was isolated in a high purity (>95%) and conversion yield of 90% in a system containing dried *tert*-amyl alcohol. Several factors were investigated in depth, with the effect on the performance of the acylation reaction. The optimal conditions seemed to be a lipase amount of 12 g/L, a temperature of 60 °C and molecular sieves of 4 Å, which amounted to 166 g/L [31].

Ma’s group continued their work on bamboo-leaf flavonoid lipophilization in order to enhance glycosylated flavonoids’ inhibitory activity toward the formation of acrylamide in a lipidic food system. In China, the antioxidant of bamboo leaves as a natural food additive was approved in 2007; it has also been reported that it can effectively reduce acrylamide formation in model food systems. The antioxidant extract from bamboo-leaves, which consist of 80% (*w*/*w*) flavonoids (two pairs of position isomers-13% orientin, 39% isoorientin, 5% vitexin and 22% isovitexin), was enzymatically acylated with lauric acid (Figure 3b). Results showed that 0.05% and 0.1% solution of acylated antioxidant extract significantly reduced the content of acrylamide in potato crisps by 44.5%, and 46.9%, respectively and this extract was more efficient for inhibiting acrylamide formation than the non-modified. According to the authors, bamboo-leaf flavonoid esters scavenged reactive carbonyls formed via Maillard reactions responsible for acrylamide formation in foods [32].

Moreover, it should be pointed out that Ma et al. [32] did not show significant concentration- and anti-concentration-dependent relationships in different ranges of treatments [32]. This phenomenon was described as an example of the “polar paradox” which had been first reported in 1989. The lipophilic antioxidants tend to be more effective in the emulsion or liposome system and less effective in bulk dry oils. The reverse is generally true for the more polar antioxidants (or amphiphiles with higher hydrophile–lipophile balance). Thus, in the members of a homologous series, a reciprocal relationship has been shown. The lower (less alkylated) members tend to be more active in dry oils and the higher members more active in emulsions [33]. Leaves of polyphenolic extracts used for their lipophilization are summarized in Table 2.

## 5. Acylation of Other Plant Derived Matrices Rich in Phenolic Compounds

In the flowering plant family, *Lamiaceae*, which includes widely used culinary and medicinal herbs, Stachys is one of the largest genera. Two Greek endemic plants, *Stachys swainsonii* ssp. argolica (Boiss.) Phitos and Damboldt and *St. swainsonii* ssp. Swainsonii, were used by Mellou et al. [29] for the preparation of lipophilic antioxidant agents. Flavonoid glycosides, namely, chrysoeriol-7-*O*-β-D-(3″-*E-p*-coumaroyl)-glucopyranoside and chrysoeriol-7-[6‴-*O*-acetyl-β-D-allosyl-(1→2)-β-D-glucopyranoside] (Figure 3c,d), were isolated from the purified methanol extracts of the aerial parts of two mentioned Greek plants and were acylated with vinyl laurate as acylation agent with immobilized *Candida antarctica* lipase B as a biocatalyst [29].

Based on the model reaction, acylation of naringin, the influence of the molar ratio of the flavonoid to acyl donor, its nature (in the form of lauric acid or its vinyl ester) and the reaction solvent (acetone or *tert*-butanol), were evaluated. Vinyl laurate, acetone and 10-fold excess of acyl donor proved to be the right choice to achieve a high conversion yield. Moreover, the used lipase showed regiospecificity for the primary alcohol of the glucose residue of tested flavonoid glycosides. Purified flavonoid esters and their precursors were compared in their ability to impact on the resistance of isolated LDL (low-density lipoprotein) and total serum to copper-induced oxidation. In line with the predictions, the use of the more lipophilic compounds caused higher prolongation of LDL and serum resistance to oxidation. Despite that, LDL oxidation is a complex process and has not yet been fully understood; the provided research definitely exhibited the antioxidant capacity of esterified flavonoids [29].

Proanthocyanidins, which are oligomeric flavonoids, have also become the subject of research on the possibility of lipophilization of polyphenols. A dimeric compound (PA) used in the study of Xiao et al. [17] was isolated from the bark of *Acacia mearnsii* and was a heteroduplex composed of procyanidin and prorobinetinidin linked by a single C4–C8 bond. The reaction conditions for the acylation of PA with palmitic acid (Figure 3e) were optimized by choosing temperature, reaction time, amount of enzyme, the molar ratio of substrates and solvent together with the initial water content.

A conversion of 97% was achieved with the following conditions: 10-fold excess of palmitic acid, *tert*-amyl alcohol with 5% of water at 60 °C for 12 h with 30 g/L of CALB. As in the earlier quoted papers, the incorporation of fatty acid into a hydrophilic molecule resulted in a significant improvement in lipophilicity. The partition coefficient of mono-acylated PA was about 2.4 times higher in comparison with the non-esterified precursor. Furthermore, the antioxidant activity of the obtained compound, its precursor and vitamin E (as a control) were compared in the DPPH radical method, where half-maximal inhibitory concentrations (IC_50_) were determined. Surprisingly, IC_50_ values for PA and its acylated derivative were 8.20 µmol/L and 5.20 µmol/L, respectively. The authors suggested that the provided research may be a way to obtain lipophilic antioxidants for possible application in the food and cosmetic industries, due to the abundance of procyanidin-prorobinetinidin heterodimers in *A. mearnsii* bark and the ease of their enzymatic acylation [17].

Coffee pulp is rich in chlorogenic acid (5-cafeoylquinic acid, 5-CGA), which has been found to be its major constituent (31–42%) [30]. The functionalization of chlorogenic acid has been proposed by enzymatic esterification of the carboxylic acid moiety of quinic acid with fatty alcohol in order to lipophilize the molecule [34]. An alternative and more efficient chemo-enzymatic strategy has also been proposed, where 5-CGA was first chemically esterified with methanol and then transesterified with fatty alcohols [35]. The lipase-catalyzed esterification of 5-CGA present in coffee pulp with 1-heptanol/pentanol/geraniol (Figure 3f) in supercritical carbon dioxide/t-butanol has been optimized, reaching an 85/82/77% conversion yield [30].

The main purpose of anthocyanins acylation is to improve their antioxidant activity and thermostability. It has been shown that acylation with aromatic acids has a particular impact on the stability of the obtained compounds due to the intramolecular co-pigmentation caused by π-π interactions [14]. In this study, the novel acylated cyanidin glycoside derivatives were obtained through the enzymatic synthesis in *tert*-butanol. Cyanidin-3-glucoside, cyanidin-3-rutinoside, and cyanidin-3,5-diglucoside were successfully extracted from underutilized plant sources including purple corn (*Zea mays*), tiliapo (*Sideroxylon palmeri*) and plum (*Prunus domestica*) husks. Acylated anthocyanins were synthesized, but only cyanidin-3-(6″-dihydroferuloyl)glucoside and cyanidin-3-(6″-dihydrosinapoyl)glucoside exhibited better thermostability than cyanidin-3-glucoside, and its antioxidant activity was improved with dihydrosinapoyl and cinnamoyl residues [14]. Similarly, Yan et al. [36] acylated anthocyanin from black rice with aromatic acid methyl ester of benzoic acid as acyl donors and CALB presence. Cyanidin 3-(6″-benzoyl)-glucoside, cyanidin 3-(6″-salicyloyl)-glucoside and cyanidin 3-(6″-cinnamoyl)-glucoside were successfully synthesized and their thermostability and light-resistivity were improved [36].

## 6. Other Unconventional Uses of Polyphenols Lipophilization

Both examples relate to the use of acylation reactions to simultaneously purify and increase the durability of camellia seed oil.

Tea is one of the most popular beverages consumed around the globe and prepared from the leaves of *Camellia sinensis.* The beneficial effect of tea on human health is primarily due to its high antioxidant activity, which results mainly from the presence of tannins and catechins (epicatechin, epigallocatechin), as well as their derivatives (epicatechin gallate and epigallocatechin gallate) [37,38,39]. Seeds are another product in addition to the leaves of the tea plant; they are the source of vegetable oil, an important plant oil due to its high content of unsaturated fatty acids, especially essential linoleic acid. However, crude camellia seed oil contains some free fatty acids, which must be removed to obtain an oil of acceptable quality. In crude camellia seed oil, the main free fatty acids were found to be oleic acid (C18:1), palmitic acid (C16:0), stearic acid (C18:0) and linoleic acid (C18:2). Chen et al. [39] reduced the free fatty acid content by lipophilization of epicatechin with these free fatty acids catalyzed by *Candida antarctica* lipase B (Novozym 435). Epicatechin fatty acid esters were thus formed and the presence of these compounds enhanced the oxidative stability of the oil at the same time [39].

Similarly, Luo et al. [40] used anthocyanins found in blueberry extract to add to crude camellia seed oil and lipophilized with free fatty acids in the oil through an enzymatic process catalyzed by *Candida antarctica* lipase B. Lipophilized anthocyanin derivatives were synthesized, which improved the oxidative stability of the oil under high-temperature treatment and observed that content of free fatty acids of the crude camellia seed oil was decreased [40]. The extracts mentioned in both Section 5 and Section 6 are collated in Table 3.

## 7. Conclusions

Different plants have the potential to be important sources of phenolic compounds which can be used as additives in food products. It should be outlined that the majority of studies focused on anthocyanins and other flavonoids. Presented studies pointed out that modified derivatives of polyphenols were more lipophilic than the native compounds and exhibited good antioxidant properties, and often higher thermal and light stability. Some acylated phenolics improved the stability of edible oils. This fact caused a positive impact on products that were fried in modified oils by increasing their nutrient values or decreasing the content of hazardous acrylamide. In the future, lipophilic compounds can be used as a possible alternative for synthetic antioxidants such as butylated hydroxytoluene (BHT), butylated hydroxyanisole (BHA) and *tert*-butylhydroquinone (TBHQ). Still, toxicological and functional research is needed to confirm that the derivatives of phenolics are safe for human health. Moreover, the outstanding issue remains, the impact of the lipophilization reaction on antioxidant properties of many esterified phenolics compared to non-modified compounds, which could be double. The reason for those observations is still unknown, and it should be explained in the future with the use of computational studies and in-depth experiments.

## Figures and Tables

**Table 1:** Plant extracts from fruits used in enzymatic modifications of phenolic compounds.

The Origin of the Plant Extract	Main Components of the Extract	Used Enzyme	Reaction Conditions	The Obtained Ester(s)	Research Highlights	Reference
Rowan (*Sorbus aucuparia* L.)	Chlorogenic acid	*Candida antarctica* lipase B	55 °C, 120 h, 2-methyl-2-butanol as a solvent, molar ratio of 1:20 (acid:alcohol)	Octadecyl chlorogenate	(a) A 43% decrease in peroxide value of rapeseed oil after fortifying with octadecyl chlorogenate. (b) Inhibition of triacylglycerols polymerization and polar compound formation during potato chip frying due to the addition of lipophilized extract.	[10]
Alpine bearberry (*Arctostaphylos alpina*)	Cyanidin-3-*O*-galactoside	*Candida antarctica* lipase B	60 °C, 72 h, *tert*-butanol as a solvent and the molar ratio of anthocyanin to lauric acid was 1:10	Cyanidin-3-*O*-(6″-dodecanoyl)galactoside	(a) Improved thermostability of the obtained ester. (b) Improved lipophilicity (higher logP value).	[11]
Blackcurrant (*Ribes nigrum* L.)	Delphinidin-3-*O*-rutinoside, Cyanidin-3-*O*-rutinoside, Delphinidin-3-*O*-glucoside, Cyanidin-3-*O*-glucoside	*Candida antarctica* lipase B	60 °C, 9 h, acetonitrile:DMSO 10:1 (*v*/*v*), 20 g/L of enzyme, molecular sieves (100 g/L), a anthocyanins:octanoic acid ratio of 1:100	Cyanidin-3-*O*-(6″-octanoyl)glucoside, Delphinidin-3-*O*-(6″-octanoyl)glucoside	(a) Lipophilization as a new anthocyanin separation technique with different glycosidic moieties.	[18]
Blackcurrant (*Ribes nigrum* L.)	Delphinidin-3-*O*-rutinoside, Cyanidin-3-*O*-rutinoside, Delphinidin-3-*O*-glucoside, Cyanidin-3-*O*-glucoside	*Candida antarctica* lipase B	60 °C, 72 h, *tert*-butanol as a solvent and the molar ratio of anthocyanin to lauric acid was 1:10, 10 g/L of enzyme, molecular sieves (100 g/L)	Cyanidin-3-*O*-(6″-dodecanoyl)glucoside, Delphinidin-3-*O*-(6″-dodecanoyl)glucoside, Cyanidin-3-*O*-(6″-dodecanoyl)rutinoside, Delphinidin-3-*O*-(6″-dodecanoyl)rutinoside,	(a) Improved thermostability due to the enzymatic acylation. (b) Acylation significantly improved inbitition of lipid peroxidation in the β-carotene bleaching method.	[19]
Trueno (*Ligustrum japonicum*) fruits	Cyanidin-3-*O*-rutinoside	*Candida antarctica* lipase B	60 °C, 48 h, 300 rpm, 20 g/L of enzyme, *tert*-butanol as a solvent, a cyanidin:vinyl cinnamate ratio of 1:250, molecular sieves (100 g/L)	Cyanidin-3-*O*-(4‴-cinnamoyl)rutinoside	(a) Optimized synthesis methodology with 45.5% conversion yield.	[14]
Raspberry (*Rubus* L.)	Cyanidin-3-*O*-glucoside	Novozym 435 (*Candida antarctica* lipase B)	40 °C, 24 h, pyridine (10 mL), 0.9 KPa of pressure (rotary evaporator), 10 mg of purified anthocyanins, 500 mg of methyl salicylate, 200 mg of Novozym 435	Cyanidin-3-(6-salicyloyl)glucoside	(a) Acylation improved thermostability and stability in the light and oxidation environments.	[12]
Penglai apple polyphenolic extract	Phloridzin	Novozym 435 (*Candida antarctica* lipase B)	60 °C, 168 h, 20 mg of fruit extract, acyl donor substrate (2 molar equivalents), 100 mg of enzyme, 100 mg of molecular sieves, *tert*-butanol as a solvent (1 mL, containing 0.2% of BHT)	Phloridzyl palmitate, Phloridzyl 4-hydroxyphenylpropionate, Phloridzyl 2-hydroxyphenylpropionate, Phloridzyl 3,4-dihydroxyphenylpropionate, phloridzyl cinnamate, Phloridzyl 3-phenylpropionate	(a) Acylation improved the solubility of the obtained derivatives in the reaction solvent.	[20]
Blueberry extract	Chlorogenic acid, Quercetin-3-glycosides, Delphinidin, cyanidin, petunidin and malvidin glycosides	Novozym 435 (*Candida antarctica* lipase B)	60 °C, 168 h, 20 mg of fruit extract, acyl donor substrate (2 molar equivalents), 100 mg of enzyme, 100 mg of molecular sieves, *tert*-butanol as a solvent (1 mL, containing 0.2% of BHT)	3-phenylpropionate esters of quercetin, isoquercetin, and delphinidin, cyanidin, petunidin and malvidin glycosides	(a) Novozym 435 showed selectivity for a primary aliphatic hydroxyl group of the sugar moiety in the acylation of different groups of polyphenols.	[20]
Canadian crabapple	Phloridzin	*Candida antarctica* lipase B	55 °C, 120 h, 250 rpm, 2-methyl-2-butanol (10 mL) as a solvent, 1000 mg of enzyme, molecular sieves (1000 mg), phenolic extract (750 mg), octadecanoic acid (acyl donor, 1500 mg)	Phloridzyl octadecanoate	(a) Improved stability of the rapeseed oil during potato chip frying (inhibition of polymerization of triacylglycerols and polar components formation). (b) Higher amount of tocopherols presented in deep fried potato chips in the oil with the addition of phloridzyl octadecanoate.	[13]
Grape seeds (GSP)	Epicatechin, Procyanidin B1, and other 9 phenolic compounds	Lipozyme TL IM (immobilized lipase from *Thermomyces lanuginosus*)	45 °C, 22 h, a ratio of lauric acid:grape seeds extract of 1:1, enzyme (2%), ethanol as a solvent	Lauroyl epicatechin, Tri-lauroyl epicatechin gallate, Lauroyl catechin	(a) GSP derivatives had the highest DPPH· scavenging activity compared to GSP, BHT (butylated hydroxytoluene) and TBHQ (*tert*-butylhydroquinone)	[21]
Skin of jaboticaba (*Myrciaria cauliflora*) fruits	Delphinidin-3-*O*-glucoside and Cyanidin-3-*O*-glucoside	Novozym 435 (*Candida antarctica* lipase B)	50 °C, 48 h, 600 rpm, 200 mbar, 20 g/L of enzyme, 20 mg of jaboticaba extract, palmitic acid as an acyl donor (2 molar equivalents), 2-methyl-2-butanol as a solvent (5 mL)	Delphinidin-3-*O*-(6″-palmitoyl)glucoside, Cyanidin-3-*O*-(6″-palmitoyl)glucoside	(a) Acylation increased the hydrophobicity of anthocyanins.	[23]
Jambolan (*Syzygium cumini*) fruits	Anthocyanins	*Candida antarctica* lipase B	40 °C, 48 h, acetone with 10% of DMSO as solvents, vinyl cinnamate as an acyl donor	Cinnamate esters of anthocyanins	(a) Higher thermal and light stability in the acylated anthocyanins compared to the native anthocyanins.	[25]

**Table 2:** Plant extracts from flowers and leaves used in enzymatic modifications of phenolic compounds.

The Origin of the Plant Extract	Main Components of the Extract	Used Enzyme	Reaction Conditions	The Obtained Ester(s)	Research Highlights	Reference
*Hibiscus sabdariffa* flowers	Delphinidin 3-*O*-sambubioside	*Candida antarctica* lipase B	60 °C, 48 h, 20 g/L of enzyme, 2-methyl-2-butanol as a solvent, molecular sieves (100 g/L), a anthocyanin:octanoic acid ratio of 1:250	Octanoic acid ester of delphinidin-3-*O*-sambubioside	(a) Stable quinoidal base with blue color at a wide range of pH.	[16]
Bottlebrush (*Callistemon citrinus*) flowers	Cyanidin-3,5-*O*-diglucoside	*Candida antarctica* lipase B	60 °C, 48 h, 300 rpm, 20 g/L of enzyme, *tert*-butanol as a solvent, a cyanidin:vinyl cinnamate ratio of 1:250, molecular sieves (100 g/L)	Cyanidin-3-*O*-(6″-cinnamoyl) glucoside-5-*O*-glucoside	(a) Optimized synthesis methodology with 85.7% conversion yield.	[14]
Rose petals	Cyanidin-3,5-*O*-diglucoside	Fermase CALB™10000 (lipase B from *Candida antarctica* immobilized on polyacrylate beads)	40 °C, 24 h, acetonitrile as a solvent, 20 mg/mL of enzyme, a cyanidin:lauric acid molar ratio of 1:100, molecular sieves (100 mg/mL)	Lauryl monoesters of cyanidin-3,5-*O*-diglucoside	(a) Enhanced color stability in thermal processing of rice extrudates. (b) The use of esterified anthocyanins as a colorant in cupcakes and as a filling in sandwich biscuit cream.	[15]
Bamboo leaves	Isoorientin and Isovitexin	*Candida antarctica* lipase B	65 °C, 48 h, molecular sieves (100 mg/mL), 10 g/L of enzyme, 2-methyl-2-butanol as a solvent, the acyl donor/flavonoid molar ratio of 5:1 or 10:1	Isoorientin-6″-laurate, Isovitexin-6″-laurate	(a) The lipophilicity was improved due to the acylation, and simultaneously, reduction in the antioxidant activity was observed.	[28]
Bamboo leaves	Isoorientin	Novozym 435 (*Candida antarctica* lipase B)	60 °C, 48 h, 210 rpm, molecular sieves (140 mg/mL), 12 g/L of enzyme, 2-methyl-2-butanol as a solvent, the acyl donor/flavonoid molar ratio reached of 12:1	Isoorientin-6″-palmitate	(a) High yield of the acylation (90%). (b) Acylation improved lipophilicity, but the antioxidant activity of the obtained derivative was lower.	[31]
Bamboo leaves	Orientin, Isoorientin, Vitexin, and Isovitexin	Novozym 435 (*Candida antarctica* lipase B)	65 °C, 48 h, 2-methyl-2-butanol as a solvent (100 mL), bamboo leaves extract (2.5 g–4.5 mM of falvonoids), 4.51 g of lauric acid (22.5 mM), a ratio of acid:extract of 5:1, 10 g of enzyme, 100 mg/mL of molecular sieves	Orientin-6″-laurate, Vitexin-6″-laurate, Isoorientin-6″-laurate, Isovitexin-6″-laurate	(a) Acylated antioxidants from bamboo leaves in the concentration of 0.05% and 0.1% inhibited the formation of acrylamide during potato chip frying.	[32]

**Table 3:** Other plant extracts used in enzymatic modifications of phenolic compounds.

The Origin of the Plant Extract	Main Components of the Extract	Used Enzyme	Reaction Conditions	The Obtained Ester(s)	Research Highlights	Reference
Aerials parts of two Greek endemic plants, i.e., *Stachys swainsonii* ssp. argolica (Boiss.) Phitos and Damboldt and *St. swainsonii* ssp. swainsonii	Chrysoeriol-7-*O*-β-D-(3″-*E-p*-coumaroyl) glucopyranoside and Chrysoeriol-7-[6‴-*O*-acetyl-β-D-allosyl-(1→2)-β-D-glucopyranoside]	Novozym 435 (*Candida antarctica* lipase B)	50 °C, 96 h, 240 rpm, acetone as a solvent (10 mL), flavonoids–0.2 mmol, vinyl laurate as an acyl donor (2 mmol, ratio 1:10), 100 mg of enzyme	Laurate ester of chrysoeriol-7-*O*-β-D-(3″-*E*-*p*-coumaroyl)-glucopyranoside and laurate ester of chrysoeriol-7-[6‴-*O*-acetyl-β-D-allosyl-(1→2)-β-D-glucopyranoside]	(a) The obtained laurate esters caused higher prolongation of LDL and serum resistance to copper-induced oxidation.	[29]
Purple corn (*Zea mays*) or tiliapo (*Sideroxylon palmeri*) husks	Cyanidin-3-*O*-glucoside	*Candida antarctica* lipase B	60 °C, 48 h, 300 rpm, 20 g/L of enzyme, *tert*-butanol as a solvent, a cyanidin:vinyl cinnamate ratio of 1:250, molecular sieves (100 g/L)	Cyanidin-3-*O*-(6″-cinnamoyl) glucoside, Cyanidin-3-*O*-(6″-dihydro cinnamoyl)glucoside, Cyanidin-3-*O*-(6″-dihydro feruloyl)glucoside, Cyanidin-3-*O*-(6″-dihydro sinapoyl)glucoside	(a) Cyanidin-3-*O*-(6″-dihydrosinapoyl) glucoside had the highest antioxidant activity in DPPH^•^ assay.	[14]
Plum (*Prunus domestica*) husk	Cyanidin-3-*O*-rutinoside	*Candida antarctica* lipase B	60 °C, 48 h, 300 rpm, 20 g/L of enzyme, *tert*-butanol as a solvent, a cyanidin:vinyl cinnamate ratio of 1:250, molecular sieves (100 g/L)	Cyanidin-3-*O*-(4‴-cinnamoyl)rutinoside	(a) Optimized synthesis methodology with a 45.5% conversion yield.	[14]
Black rice (*Oryza sativa* L. subsp. *Japonica*)	Cyanidin-3-*O*-glucoside	Novozym 435 (*Candida antarctica* lipase B)	40 °C, 48 h, 30 rpm, 900 mbar (vacuum pump), 0.5 g of black rice anthocyanins, 10 mL of acyl donor, pyridine (5 mL) as a solvent, 1 g of enzyme	Cyanidin-3-*O*-(6″-benzoate) glucoside, Cyanidin-3-*O*-(6″-salicylate) glucoside, Cyanidin-3-*O*-(6″-cinnamate) glucoside	(a) Enzymatic acylation improved the half-life times of anthocyanins in the light treatments (dark, UV and fluorescent). (b) Improved thermostability due to the enzymatic acylation was observed.	[36]
*Acacia mearnsii* bark	Heteroduplex composed of procyanidin and prorobinetinidin linked by a single C4–C8 bond (PA dimers)	Novozym 435 (*Candida antarctica* lipase B)	60 °C, 12 h, 30 g/L of enzyme, 2-methyl-2-butanol (with 5% of water) as a solvent, a ratio of 10:1 (palmitic acid:PA dimers)	Procyanidin and prorobinetinidin heteroduplex esterified with palmitic acid	(a) The antioxidant activities of PA dimers and obtained palmitate ester were much higher than the activity of vitamin E.	[17]
Coffee pulp	Chlorogenic acid	Novozym 435 (*Candida antarctica* lipase B)	Cell volume of 50 mL, 150 mbar of supercritical carbon dioxide (sCO_2_), 55 °C, 25 h, *tert*-butanol as a solvent (10% *v*/*v*), 10% (*v*/*v*) of acyl donor (1-heptanol or 1-pentanol or geraniol), 20 mg/mL of enzyme, 20 mg/mL of molecular sieves	Heptyl chlorogenate	(a) The supercritical carbon dioxide proved to be useful in the lipophilization of chlorogenic acid from the coffee pulp.	[30]
Tea leaves	Epicatechin	Novozym 435 (*Candida antarctica* lipase B)	50 °C, 36 h, 14 mg of epicatechin (dissolved in polyoxyethylene stearate, 1:1 (m/m)), 10 g of crude camellia seed oil, 10 mg of enzyme, 2 g of molecular sieves	Epicatechin palmitate, Epicatechin oleate	(a) Reduced content of the free fatty acids as a result of the reaction of blueberry anthocyanins with components of the crude camellia seed oil. (b) The oxidative stability of the oil was improved.	[39]
Blueberry anthocyanin extract	Malvidin-3-*O*-galactoside, Cyanidin-3-*O*-galactoside, Delphinidin-3-*O*-galactoside, Malvidin-3-*O*-glucoside, and Cyanidin-3-*O*-arabinoside	Novozym 435 (*Candida antarctica* lipase B)	50 °C, 36 h, 200 rpm, 36 mg of the blueberry anthocyanin solution (dissolved in polyoxyethylene stearate, 1:2 (m/m)), 10 g of crude camellia seed oil, 10 mg of enzyme, 2 g of molecular sieves	Cyanidin-3-*O*-(6″-oleoyl) galactoside, Cyanidin-3-*O*-(6″-palmitoyl) galactoside	(a) Reduced content of the free fatty acids as a result of the reaction of blueberry anthocyanins with components of the crude camellia seed oil. (b) The oxidative stability of the oil was improved.	[40]
